# Using Topic Modeling Methods for Short-Text Data: A Comparative Analysis

**DOI:** 10.3389/frai.2020.00042

**Published:** 2020-07-14

**Authors:** Rania Albalawi, Tet Hin Yeap, Morad Benyoucef

**Affiliations:** ^1^School of Information Technology and Engineering, University of Ottawa, Ottawa, ON, Canada; ^2^Telfer School of Management, University of Ottawa, Ottawa, ON, Canada

**Keywords:** natural language processing, topic modeling, short text, user-generated content, online social networks

## Abstract

With the growth of online social network platforms and applications, large amounts of textual user-generated content are created daily in the form of comments, reviews, and short-text messages. As a result, users often find it challenging to discover useful information or more on the topic being discussed from such content. Machine learning and natural language processing algorithms are used to analyze the massive amount of textual social media data available online, including topic modeling techniques that have gained popularity in recent years. This paper investigates the topic modeling subject and its common application areas, methods, and tools. Also, we examine and compare five frequently used topic modeling methods, as applied to short textual social data, to show their benefits practically in detecting important topics. These methods are latent semantic analysis, latent Dirichlet allocation, non-negative matrix factorization, random projection, and principal component analysis. Two textual datasets were selected to evaluate the performance of included topic modeling methods based on the topic quality and some standard statistical evaluation metrics, like recall, precision, *F*-score, and topic coherence. As a result, latent Dirichlet allocation and non-negative matrix factorization methods delivered more meaningful extracted topics and obtained good results. The paper sheds light on some common topic modeling methods in a short-text context and provides direction for researchers who seek to apply these methods.

## Introduction

People nowadays tend to rely heavily on the internet in their daily social and commercial activities. Indeed, the internet has increased demand for the development of commercial applications and services to provide better shopping experiences and commercial activities for customers around the world. The internet is full of information and sources of knowledge that may confuse readers and cause them to spend additional time and effort in finding relevant information about specific topics of interest. Consequently, there is a need for more efficient methods and tools that can aid in detecting and analyzing content in online social networks (OSNs), particularly for those using user-generated content (UGC) as a source of data. Furthermore, there is a need to extract more useful and hidden information from numerous online sources that are stored as text and written in natural language within the social network landscape (e.g., Twitter, LinkedIn, and Facebook). It is convenient to employ a natural approach, similar to a human–human interaction, where users can specify their preferences over an extended dialogue.

Natural language processing (NLP) is a field that combines the power of computational linguistics, computer science, and artificial intelligence to enable machines to understand, analyze, and generate the meaning of natural human speech. The first actual example of the use of NLP techniques was in the 1950s in a translation from Russian to English that contained numerous literal transaction misunderstandings (Hutchins, 2004). Essentially, keyword extraction is the most fundamental task in several fields, such as information retrieval, text mining, and NLP applications, namely, topic detection and tracking (Kamalrudin et al., 2010). In this paper, we focused on the topic modeling (TM) task, which was described by Miriam (2012) as a method to find groups of words (topics) in a corpus of text. In general, the procedure of exploring data to collect valuable information is stated as text mining. Text mining includes data mining algorithms, NLP, machine learning, and statistical operations to derive useful content from unstructured formats such as social media textual data. Hence, text mining can improve commercial trends and activities by extracting information from UGC.

**Figure 1:**
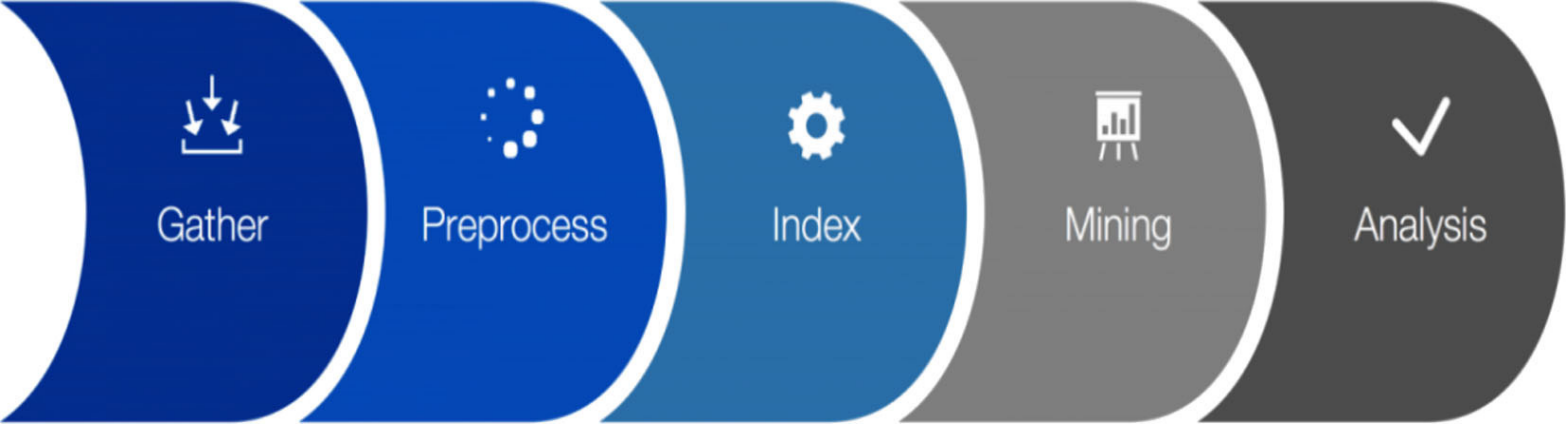
The steps involved in a text mining process (Kaur and Singh, 2019).

TM methods have been established for text mining as it is hard to identify topics manually, which is not efficient or scalable due to the immense size of data. Various TM methods can automatically extract topics from short texts (Cheng et al., 2014) and standard long-text data (Xie and Xing, 2013). Such methods provide reliable results in numerous text analysis domains, such as probabilistic latent semantic analysis (PLSA) (Hofmann, 1999), latent semantic analysis (LSA) (Deerwester et al., 1990), and latent Dirichlet allocation (LDA) (Blei et al., 2003). However, many existing TM methods are incapable of learning from short texts. Also, many issues exist in TM approaches with short textual data within OSN platforms, like slang, data sparsity, spelling and grammatical errors, unstructured data, insufficient word co-occurrence information, and non-meaningful and noisy words. For example, Gao et al. (2019) discussed the problem of word sense disambiguation by using local and global semantic correlations, achieved by a word embedding model. Yan et al. (2013) developed a short-text TM method called biterm topic model (BTM) that uses word correlations or embedding to advance TM. The fundamental steps involved in text mining are shown in Figure 1, which we will explain later on our data preprocessing step.

In general, TM has proven to be successful in summarizing long documents like news, articles, and books. Conversely, the need to analyze short texts became significantly relevant as the popularity of microblogs, such as Twitter, grew. The challenge with inferring topics from short text is that it often suffers from noisy data, so it can be difficult to detect topics in a smaller corpus (Phan et al., 2011).

This paper makes the following contributions:

- We review scholarly articles related to TM from 2015 to 2020, including its common application areas, methods, and tools.
- We investigate select TM methods that are commonly used in text mining, namely, LDA, LSA, non-negative matrix factorization (NMF), principal component analysis (PCA), and random projection (RP). As there are many TM methods in the field of short-text data, and all definitely cannot be mentioned, we selected the most significant methods for our work.
- We evaluate all included TM methods based on two dimensions, the understandability of extracted topics (topic quality) besides the topic performance and accuracy by applying common standard metrics that apply to the TM domain such as recall, precision, *F*-score, and topic coherence. In addition, we consider two textual datasets: the 20-newsgroup data, common for evaluations in social media text application tasks, and 20 short conversation data from Facebook, a popular social network site.
- We aim to compare and evaluate many TM methods to define their effectiveness in analyzing short textual social UGC.

The paper is organized as follows. Section Literature Review contains a comprehensive summary of some recent TM surveys as well as a brief description of the related subjects on NLP, specifically the TM applications and toolkits used in social network sites. In Section Proposed Topic Modeling Methodology, we focus on five TM methods proposed in our study besides our evaluation process and its results. The conclusion is presented in section Evaluation along with an outlook on future work.

## Literature Review

To obtain a comprehensive summary of recent surveys, we started by exploring existing studies related to the area of TM for long and short texts. Additionally, we reviewed the most common TM applications, tools, and algorithms as applied to OSNs. For example, Jelisavčić et al. (2012) provided an overview of the most popular probabilistic models used in the TM area. Hong and Brian Davison (2010) compared the performance of the LDA method and author–topic models on the Twitter platform. Alghamdi and Alfalqi (2015) proposed an empirical study of TM by categorizing the reviewed works into two popular approaches: topic evolution models and standard topic models with a time factor. Song et al. (2014) presented a survey about short-text characteristics, challenges, and classification that were divided into four basic types, namely, the usage of semantic analysis, classification using semi-supervised methods, fusion-based ensemble technique, and real-time classification. Jaffali et al. (2020) presented a summary of social network data analysis, including its essential methods and applications in the context of structural social media data analysis. They structured the social network analysis methods into two types, namely, structural analysis methods (which study the structure of the social network like friendships), and added-content methods (which study the content added by users). Likhitha et al. (2019) presented a detailed survey covering the various TM techniques in social media text and summarized many applications, quantitative evaluations of various methods, and many datasets that are used with various challenges in short content and documents. Table 1 presents several related works that reviewed the TM methods in long/short textual social media data. Different from existing reviewed works, our paper not only focuses on the review of TM tools, applications, and methods but also includes several evaluations applying many techniques over short textual social media data to determine which method is the best for our future proposed system that aims to detect real-time topics from online user-generated content.

**Table 1:** Some of the existing related works that revised the topic modeling method.

**Related work**	**Topic modeling method**	**Evaluation method**	**Outcome**
Chakkarwar and Tamane (2020)	Latent Dirichlet allocation (LDA) with bag of words (BoW)	Visual overview of extracted topics	- Aimed to discover the current trends, topics, or patterns from research documents to overview different research trends.
			- The result shows that the LDA is an effective topic modeling method for creating the context of a document collection.
Ray et al. (2019)	Latent semantic indexing (LSI)	Perplexity	- Aimed to introduce methods and tools of topic modeling to the Hindi language.
	LDA	Topic coherence	- Discussed many techniques and tools used for topic modeling.
	Non-negative matrix factorization (NMF)		- The coherence result of the NMF model was a little better than the LDA model.
			- The perplexity of the LDA model on the Hindi dataset is better compared to other evaluated topic modeling methods.
Xu et al. (2019)	LDA	Perplexity	- Aimed to help Chinese movie creators to get the psychological needs of movie viewers and provide suggestions to improve the quality of Chinese movies.
			- Used the word cloud as a visual display of high-frequency keywords in a text which gives a basic understanding of the core ideas of text data.
			- The LDA model provides topics that deliver a good analysis of the Douban online review.
			- Used the perplexity method to determine the best number of extracted topics, as a result, 20 extracted topics were set.
Alghamdi and Alfalqi (2015)	Latent semantic analysis (LSA)		- Reviewed many topic modeling methods in terms of characteristics, limitations, and theoretical background.
	Probabilistic latent semantic analysis (PLSA)		
	LDA		- Reviewed many topic modeling application areas and evaluation methods.
	Correlated topic model (CTM)		
Chen et al. (2017)	NMF	*t*-Distributed stochastic neighbor embedding (TSNE) dimensionality-reduction method	- Aimed to compare and evaluate many topic modeling approaches in analyzing a large set of the US Securities and Exchange Commission (SEC) filings made by US public banks.
	Principal component analysis (PCA)		- Both NMF and LDA methods provide very good document representation, while the K-Competitive Autoencoder for Text (KATE)^1^ delivered more meaningful document and high-accuracy topics.
	LDA		
	KATE		- The LDA provided the best result regarding the classification of topic representation.
Mazarura and de Waal (2016)	LDA	Topic stability	- Tested many numbers of topics (10, 20, 30, 40, 50, and 100 topics).
			- Topic coherence decreases for both the LDA and Dirichlet multinomial mixture model (GSDMM) as the number of topics increases in a long text, which indicates an overall decline in the quality of topics uncovered by both models as the number of topics increases.
	GSDMM	Topic coherence	- The LDA's performance of the coherence values is slightly better than the GSDMM.
			- The GSDMM is more stable than LDA.
			- The GSDMM is indeed a viable option on the short text as it displays the potential to produce better results than LDA.
Sisodia et al. (2020)	BoW		- The Nu-support vector classification (Nu-SVC) classifier outperforms all other included classifiers in the set of individual classifiers.
	Term frequency–inverse document frequency (TF-IDF)	Accuracy	- Random forest classifier outperforms all other included classifiers in the set of the case on ensemble classifiers.
	Naive Bayes	Precision	- The support vector machine (SVM) classifier outperforms all other classifiers in the set of individual classifiers.
	SVM	Recall	- Random forest classifier outperforms the remaining ones.
	Decision trees	*F*-measures	- Considered only two datasets; other datasets of different sizes need to be studied for better results.
	Nu-SVC		
Shi et al. (2017)	Vector space model (VSM)		- Reviewed all of the following methods: VSM, LSI, PLSA, and LDA.
	LSI		- Reviewed the essential concept of topic modeling using a bag-of-words approach.
	PLSA		- Discussed the basic idea of topic modeling including the bag-of-words approach, training of model, and output.
	LDA		- Discussed topic modeling application, features, limitations, and tools such as Gensim, standard topic modeling toolbox, Machine Learning for Language Toolkit (MALLET), and BigARTM.
Nugroho et al. (2020)	LDA	Purity	- It focuses on the review of the approaches and discusses the features that are exploited to deal with the extreme sparsity and dynamics of the online social network (OSN) environment.
	NMF	Normalized mutual information (NMI)	- Run the algorithms over both datasets 30 times and note the average value of each evaluation metric for comparison.
	Task-driven NMF		- Most methods can achieve high purity value.
			- The NMF and non-negative matrix inter-joint factorization (NMijF) having the best performance over the other methods.
	Plink-LDA	Pairwise *F*-measure	- *F*-measure evaluation results in all methods were well and similar.
			- NMijF provides the best results according to all the evaluation metrics.
	NMijF		- Both LDA and NMF focus on the simple content exploitation of social media posts, main features (content, social interactions, and temporal).
Ahmed Taloba et al. (2018)	PCA model	Precision	- The aim was to compare the performance of these methods before and after using PCA.
	Standard SVM	Accuracy	
	J-48 decision tree	Sensitivity	- The RF gives acceptable and higher accuracy when compared to the rest of the classifiers.
	KNN methods	*F*-measure	- The RF algorithm gives higher performance, and its performance is improved after using PCA.
Chen et al. (2019)	LDA	PMI score	- Tested many numbers of topics (20, 40, 60, 80, and 100).
			- The NMF has overwhelming advantages over LDA.
	NMF	Human judgments	- The knowledge-guided NMF (KGNMF) model performs better than NMF and LDA
	KGNMF		- The NMF provides better topics than LDA with topic numbers ranging from 20 to 100.
Anantharaman et al. (2019)	LDA	Precision	- Evaluated all topic modeling algorithms with both BoW and TF-IDF representations.
		Recall *F*-measure	- Used the Naïve Bayes classifier for the 20-newsgroup dataset and the random forest classifier for the BBC news and PubMed datasets.
	LSA	Accuracy Cohen's	- The results of the 20-newsgroup dataset LDA with BoW outperform those of the other topic algorithms.
		Kappa score	- The LDA model does not perform well with TF-IDF when compared to BoW.
	NMF	Matthews	
		Correlation coefficient	- The LDA takes a lot of time when compared to the LSA and NMF models.
		Time taken	

1 *https://github.com/hugochan/KATE*.

In recent years, most of the data in every sphere of our lives have become digitized, and as a result, there is a need for providing powerful tools and methods to deal with this existing digital data increase in order to understand it. Indeed, there have been many developments in the NLP domain, including rule-based systems and statistical NLP approaches, that are based on machine learning algorithms for text mining, information extraction, sentiment analysis, etc. Some typical NLP real-world applications currently in use include automatically summarizing documents, named entity recognition, topic extraction, relationship extraction, spam filters, TM, and more (Farzindar and Inkpen, 2015). In the areas of information retrieval and text mining, such as the TM method, several methods perform keyword and topic extraction (Hussey et al., 2012). TM is a machine learning method that is used to discover hidden thematic structures in extensive collections of documents (Gerrish and Blei, 2011).

TM is a challenging research task for short texts, and several methods and techniques have been proposed to solve the lack of contextual information. Numerous proposed methods are established on the generative probabilistic model such as the LDA TM. In this paper, we aim to understand the real meaning of a given text, not just to extract a list of related keywords. To achieve this, we first need to understand and have a general idea about many TM methods as they can be applied in short UGC (e.g., abstract, dialogue, and Twitter text). Several TM methods are used to obtain topics from text, such as emails, documents, and blogs. The choice of technique to extract topics is based on the length of the text. For example, counting word frequencies is an appropriate method to use with a single document or a small number of documents. Liu et al. (2016) reviewed TM techniques for sentimental analysis. Meanwhile, Zihuan et al. (2018) proposed a news-topic RS based on extracting topic keywords from internet news for a specific time. They applied different keyword extraction algorithms, such as term frequency–inverse document frequency (TF-IDF) and rapid algorithm for keyword extraction (RAKE), to extract the most descriptive terms in a document. This system was efficient in obtaining a particular topic at any specific time. However, they only focused on one dataset that was about the political domain and the words that appear repeatedly; this is considered to be an issue in this recommendation system. Similarly, Shi et al. (2017) developed a semantics-assisted non-negative matrix factorization (SeaNMF) model by using a baseline of LDA and author–topic model to integrate semantic relations between word and context.

To date, the LDA model is the most popular and highly studied model in many domains and numerous toolkits such as Machine Learning for Language Toolkit (MALLET), Gensim,^1^ and Stanford TM toolbox (TMT),^2^ because it is able to address other models' limitations, such as latent semantic indexing (LSI) (Deerwester et al., 1990) and probabilistic latent semantic indexing (PLSI) (Hofmann, 2001). The LDA method can produce a set of topics that describe the entire corpus, which are individually understandable and also handle large-scale document–word corpus without the need to label any text. Keerthana (2017) developed a document recommendation system from converted text from the ASR system that used both cosine similarity (word co-occurrence) and semantic methods, as well as the LDA TM method that was implemented in the MALLET toolkit environment, to extract the most significant terms for short conversation fragments. Initially, the topic model was used to define weights for the abstract topics. After extracting the keywords, TM similarity methods were applied. In this work, researchers compared extracted keywords from different techniques, namely, cosine similarity, word co-occurrence, and semantic distance techniques. They found that extracted keywords with word co-occurrence and semantic distance can provide more relevant keywords than the cosine similarity technique.

### TM Application

TM can be applied to numerous areas like NLP, information retrieval, text classification and clustering, machine learning, and recommendation systems. TM methods may be supervised, unsupervised, or semi-supervised; may use structured or unstructured data; and may be applied in several application fields such as health, agriculture, education, e-commerce, social network opinion analysis, and transport/data network. TM can be used to discover latent abstract topics in a collection of text such as documents, short text, chats, Twitter and Facebook posts, user comments on news pages, blogs, and emails. Weng et al. (2010) and Hong and Brian Davison (2010) addressed the application of topic models to short texts. Some major application areas where researchers have used TM methods include the following:

- Recommendation systems: in many real-time systems, for example, job recommendation by mapping the right job for interested candidates based on their information, history, sociology, location, media theory, and other contexts.
- Financial analysis: in many commercial activities like structuring of the stock market exchange, using stock value information to induce subjects over diverse trades on a market organization, and other activities.
- Bioinformatics: to identify the knowledge structure of the field, e.g., study patient-related texts constructed from their clinical records.
- Manufacturing applications: used in numerous search engines, online advertising systems, and social media blogs.
- Computer science: extracting valuable information from data, image processing, and annotating images with words.
- Social network analysis (SNA): mining information about the real world in social web platforms such as inferring significant aspects about the users and services.
- Software engineering: mining unstructured repositories in the software industry such as source code, test, and bugs to support many engineering tasks like program comprehension and location (Panichella et al., 2013).

### Toolkits for Topic Models

Many TM methods and analyses are available nowadays. Below are selected toolkits that are considered standard toolkits for TM testing and evaluation.

- Stanford TMT, presented by Daniel et al. (2009), was implemented by the Stanford NLP group. It is designed to help social scientists or other researchers who wish to analyze voluminous textual material and tracking word usage. It includes many topic algorithms such as LDA, labeled LDA, and latent Dirichlet allocation (PLDA); besides, the input can be text in Excel or other spreadsheets.
- VISTopic is a hierarchical topic tool for visual analytics of text collections that can adopt numerous TM algorithms such as hierarchical latent tree models (Yang et al., 2017).
- KEA is an open-source software distributed in the Public License GNU and was used for keyphrase extraction from the entire text of a document; it can be applied for free indexing or controlled vocabulary indexing in the supervised approach. KEA was developed based on the work of Turney (2002) and was programmed in the Java language; it is a simple and efficient two-step algorithm that can be used across numerous platforms (Frank et al., 1999).
- MALLET, first released in 2002 (Mccallum, 2002), is a topic model tool written in Java language for applications of machine learning like NLP, document classification, TM, and information extraction to analyze large unlabeled text. The MALLET topic model includes different algorithms to extract topics from a corpus such as pachinko allocation model (PAM) and hierarchical LDA.
- FiveFilters is a free software tool to obtain terms from text through a web service. This tool will create a list of the most relevant terms from any given text in JSON format.
- Gensim, presented by Rehurek (2010), is an open-source vector space modeling and topic modeling toolkit implemented in Python to leverage large unstructured digital texts and to automatically extract the semantic topics from documents by using data streaming and efficient incremental algorithms unlike other software packages that only focus on batch and in-memory processing. Also, Gensim includes several kinds of algorithms such as LDA, RP, LSA, TF-IDF, hierarchical Dirichlet processes (HDPs), LSI, and singular value decomposition (SVD). Hence, all the mentioned algorithms are unsupervised, so there is no need for human input or training corpus. In addition, Gensim is considered to be faster than other topic modeling tools such as MALLET and scalable.
- Fathom provides TM of graphical visualization and calls of topic distributions (Dinakar et al., 2015).
- R TM packages include three packages that are capable of doing topic modeling analysis which are MALLET, topic models, and LDA. Also, the R language has many packages and libraries for effective topic modeling like LSA, LSAfun (Wild, 2015), topicmodels (Chang, 2015), and textmineR (Thomas Jones, 2019).
- For other open-source toolkits besides those mentioned above, David Blei's Lab provides many TM open-source software that is available in GitHub such as online inference for HDP in the Python language and TopicNets (Gretarsson et al., 2012).

## Proposed Topic Modeling Methodology

**Figure 2:**
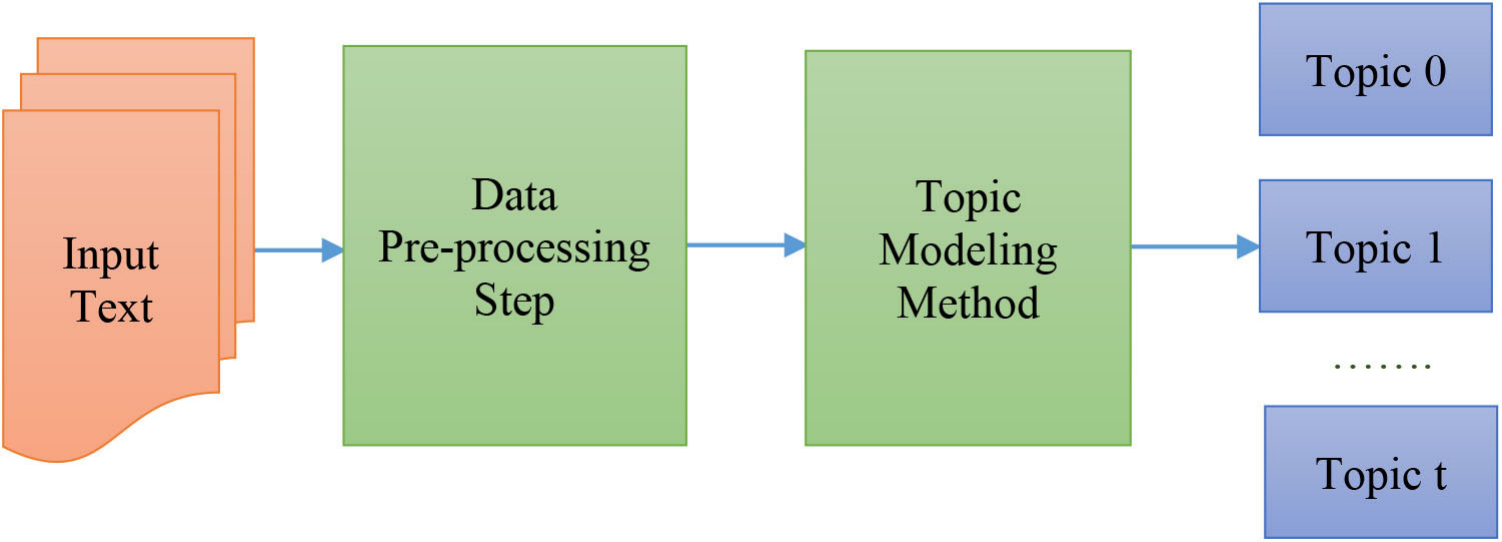
Topic modeling for text data.

TM is a methodology for processing the massive volume of data generated in OSNs and extracting the veiled concepts, protruding features, and latent variables from data that depend on the context of the application (Kherwa and Bansal, 2018). Several methods can operate in the areas of information retrieval and text mining to perform keyword and topic extraction, such as MAUI, Gensim, and KEA. In the following, we give a brief description of the included TM methods in this comparison review. In this paper, we focused on five frequently used TM methods that are built using a diverse representation form and statistical models. A standard process for topic generation is shown in Figure 2. We define the main advantages and disadvantages of all involved topic methods as shown in Table 2, and we evaluate the topic quality and performance of many TM methods; the fundamental difference among all involved methods is in how they capture the structures and in which parts of the structures they exploit. However, there are numerous TM methods used in the field of social media textual data, and as we definitely cannot mention all of them, we selected the most popular methods to compare; we then define which method is suitable to integrate in our future proposed real-time social recommendation system called ChatWithRec system (Albalawi and Yeap, 2019; Albalawi et al., 2019).

**Table 2:** Main advantages and disadvantages of the TM methods.

**TM method**	**Advantage**	**Disadvantage**
LSA	Solves the data sparsity problem and captures synonyms of words. Reduces the dimensionality of TF-IDF by using singular value decomposition. It does not require a robust statistical background and probability theory. Exploits unique structure as factors.	Difficult to label a topic in some cases and to establish a number of topics. The determination of topic numbers depends upon the human judgment and cannot be determined statistically. It does not capture the correlation between multiple topics.
LDA	It does not require any previous training data. Provides more semantically interpretable data and performs well if there is no time constraint. Handles long documents and is able to show adjectives and nouns in topics. Handles mixed-length documents. Able to enhance transitive relations between topics and obtain high-order co-occurrence in small documents like in paragraphs and sentences text.	Needs aggregation of short messages to avoid data sparsity in short documents. Unable to model relations among topics that help to understand deep structures of documents. A slow process algorithm. Requires a predefined number of topics (*T*). If *T* is too small—topics are more general if *T* is too large—topics will be overlapping with each other.
NMF	Fast process for a large amount of real-time data. Able to extract meaningful topics without prior information or knowledge of the underlying meaning in the original data. Appropriate for word and vocabulary recognition tasks.	Sometimes provides semantically incorrect results.
PCA	Low noise sensitivity and decreased need for capacity. Maintains the best possible estimate and works well on moderately low-dimensional data. It decreases the noise data because the maximum variation source is chosen and the small variations are ignored automatically. Recommended in work that aims to introduce new features by losing original features in the procedure of transformation of the high dimensions data into low dimensions. Delivers an output that can be visualized as a solid version of the main dataset.	The covariance matrix is difficult to evaluate in an accurate manner (Phillips et al., 2005). Cannot detect the simplest invariance data sometimes, unless the training data explicitly offer this information (Li et al., 2008). Expensive to compute particularly for high-dimensional datasets.
RP	Robust. Provides good results in data streaming task and if data are so high dimensional. Valid to use in imbalanced datasets. Advance linear separability. Good at discovering discriminative features.	Data sparsity. Slow predictions. Sensitive to noise data. Bad at fitting complex features. Applicable to only a few datasets.

### TM Methods

**Figure 3:**
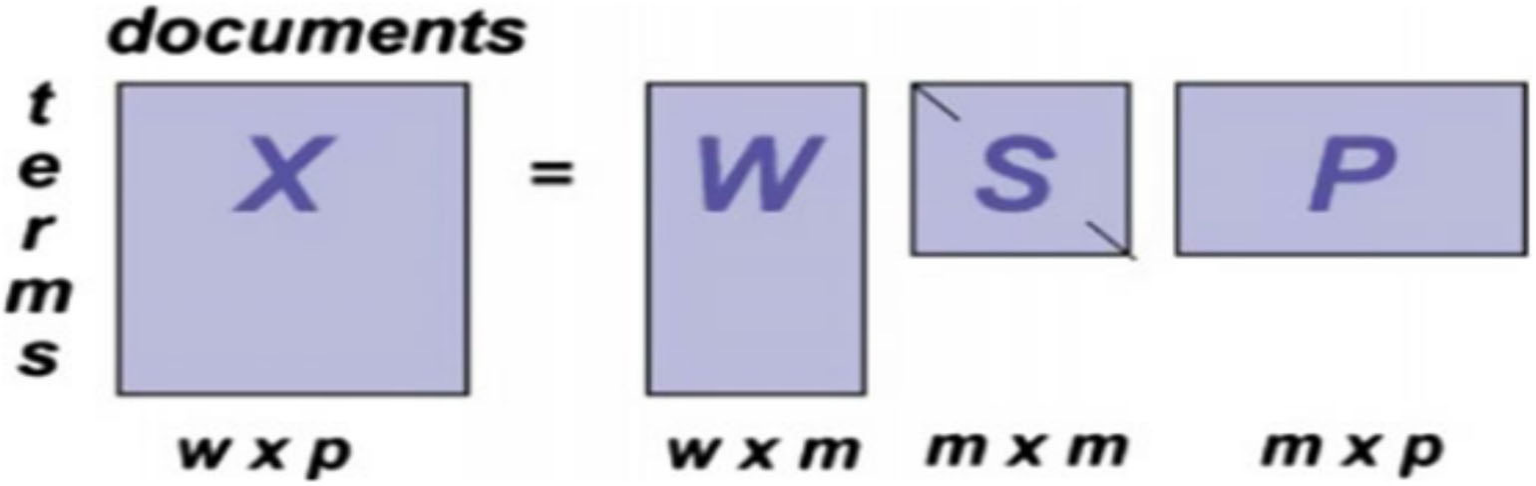
SVD of the LSA topic modeling method (Neogi et al., 2020).

**Figure 4:**
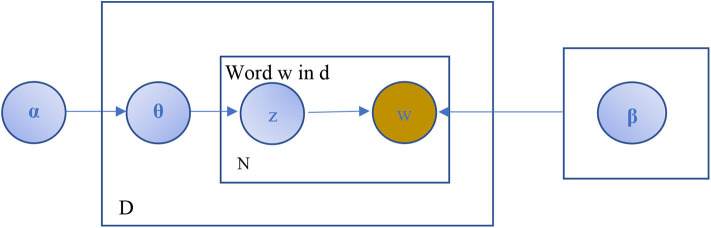
The original structure of the LDA topic model.

- LSA: It is a method in NLP proposed by Deerwester et al. (1990), particularly distributional semantics, that can be used in several areas, such as topic detection; it has become a baseline for the performance of many advanced methods. Distributional hypotheses make up the theoretical foundation of the LSA method, which states terms with similar meaning are closer in terms of their contextual usage, assuming that words that are near in their meaning show in the related parts of texts (Dudoit et al., 2002). Also, it analyzes large amounts of raw text into words and separate them into meaningful sentences or paragraphs. LSA considers both the similarity terms of text and related terms to generate more insights into the topic. Besides, the LSA model can generate a vector-based representation for texts which aids the grouping of related words. A mathematical approach called SVD is used in the LSA model to outline a base for a shared semantic vector space that captures the maximum variance across the corpus. (Neogi et al., 2020) stated that the LSA method as shown in Figure 3 learns latent topics by performing matrix decomposition on the term–document matrix; let's say X is a term-by-document matrix that decomposed into three other matrices, S, W, and P; multiplying together those matrices, we give back the matrix X with {X} = {S}{W}{P}; each paragraph is characterized by the columns, and the rows characterize the unique words. Figure 3 presents the SVD of the LSA TM method.
- LDA, introduced by Blei et al. (2003), is a probabilistic model that is considered to be the most popular TM algorithm in real-life applications to extract topics from document collections since it provides accurate results and can be trained online. Corpus is organized as a random mixture of latent topics in the LDA model, and the topic refers to a word distribution. Also, LDA is a generative unsupervised statistical algorithm for extracting thematic information (topics) of a collection of documents within the Bayesian statistical paradigm. The LDA model assumes that each document is made up of various topics, where each topic is a probability distribution over words. A significant advantage of using the LDA model is that topics can be inferred from a given collection without input from any prior knowledge. A schematic diagram of the LDA topic model is shown in Figure 4.

In Figure 4, α is a parameter that represents the Dirichlet prior for the document topic distribution, β is a parameter that represents the Dirichlet for the word distribution, θ is a vector for topic distribution over a document *d, z* is a topic for a chosen word in a document, *w* refers to specific words in *N*, plate *D* is the length of documents, and plate *N* is the number of words in the document.

**Figure 5:**
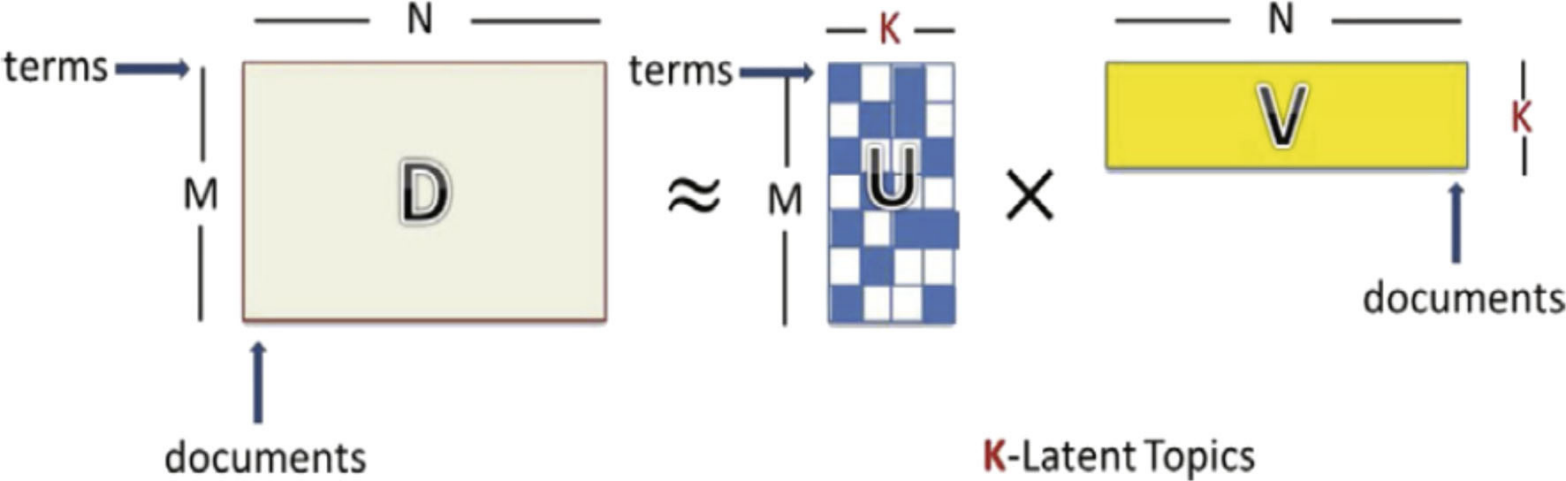
The original structure of the NMF topic model (Chen et al., 2019).

- NMF is an unsupervised matrix factorization (linear algebraic) method that is able to perform both dimension reduction and clustering simultaneously (Berry and Browne, 2005; Kim et al., 2014). It can be applied to numerous TM tasks; however, only a few works were reported to determine topics for short texts. Yan et al. (2013) presented an NMF model that aims to obtain topics for short-text data by using the factorizing asymmetric term correlation matrix, the term–document matrix, and the bag-of-words matrix representation of a text corpus. Chen et al. (2019) defined the NMF method as decomposing a non-negative matrix *D* into non-negative factors *U* and *V, V* ≥ 0 and *U* ≥ 0, as shown in Figure 5. The NMF model can extract relevant information about topics without any previous insight into the original data. NMF provides good results in several tasks such as image processing, text analysis, and transcription processes. In addition, it can handle the decomposition of non-understandable data like videos.

In Figure 5, *D* ≈ *UV*, where *U* and *V* are elementwise non-negative and, for a given text, corpus is decomposed into two matrices which are term-topic matrix U and topic–document matrix V, corresponding to K coordinate axes and N points in a new semantic space, respectively (each point represents one document).

- PCA is an essential tool for text processing tasks, and it has been used since the early 1990s (Jolliffe, 1986; Slonim and Tishby, 2000; Gomez et al., 2012). The PCA method has been used to decrease feature vector to a lower dimension while retaining the most informative features in several experimental and theoretical studies. However, it is expensive to compute for high-dimensional text datasets. The PCA TM method found a *d*-dimensional subspace of *R*^*n*^ that could capture as much of the dataset's variation as possible; specifically, given data *S* = {*x*_1_, …, *x*_m_}, we would find the linear projection to *R*^*d*^ as in Equation 1, proposed by Dasgupta (2000):
  (1) $$\begin{array}{r} \sum_{i=1}^{m}\;∥\;\chi_{i}^{*}\;-\;\mu^{*}∥{\;}^{2} \end{array}$$
  where $\chi_{i}^{*}$ is the projection of a point χ_*i*_ and μ_*_ is the mean of the projected data.
- RP has attracted attention and has been employed in many machine learning scenarios recently such as classification, clustering, and regression (Wang and McCallum, 2006; Ramage et al., 2011). The RP TM method uses a random matrix to map the original high-dimensional data onto a lower-dimensional subspace with the reduced time cost (Dasgupta, 2000). The main idea behind the RP method stems from Johnson and Lindenstrauss (1984), who states that as “a set of *n* points in a high-dimensional vector space can be embedded into *k* = ϑ(ε^−2^ log *n*) dimensions, with the distances between these points preserved up to a factor of 1+ε1+ϵ. This limit can be realized with a linear projection **Ã** = **AR**, for a carefully designed random matrix **R**ϵ ℝ^ϵ × *k*^ (*k*≪d), where **A**ϵ ℝ^n × *d*^ denote a data matrix consisting of *n* data points in ℝ^d^” (Wójcik and Kurdziel, 2019). In addition, RP has attracted lots of attention, and its accuracy for dimensionality reduction of high-dimensional datasets and directions of projection is independent of the data (does not depend on training data). Still, RP delivers sparse results because it does not consider the fundamental structure of the original data and frequently leads to high distortion.

### Data Preprocessing

In our experiment, all input data were text data that possess the English language properties. As shown in Figure 1, the first steps in the text mining process were to collect unstructured and semi-structured data from multiple data sources like microblogs and news web pages. Next, the preprocessing step was applied to clean up the data and then convert the extracted information into a structured format to analyze the patterns (visible and hidden) within the data. Extracted valuable information can be stored in a database, for example, to assist the decision-making process of an organization. Corpus preparation and cleaning were done using a series of packages running on top of Python such as the Natural Language Toolkit (NLTK) (Bird et al., 2009) that provides stop-word removal (Bird and Loper, 2004), stemming, lemmatizing, tokenization, identifying *n*-gram procedures, and other data cleanings like lowercase transformation and punctuation removal. The preprocessing steps are supported in Stanford's NLTK Library (Kolini and Janczewski, 2017; Phand and Chakkarwar, 2018) and contain the following patterns:

- Stop-word elimination: removal of the most common words in a language that are not helpful and in general unusable in text mining like prepositions, numbers, and words that do not contain applicable information for the study. In fact, in NLP, there is no particular general list of stop words used by all developers who choose their list based on their goal to improve the recommendation system performance.
- Stemming: the conversion of words into their root, using stemming algorithms such as Snowball Stemmer.
- Lemmatizing: used to enhance the system's accuracy by returning the base or dictionary form of a word.
- Tokenizing: dividing a text input into tokens like phrases, words, or other meaningful elements (tokens). The outcome of tokenization is a sequence of tokens.
- Identifying *n*-gram procedure such as bigram (phrases containing two words) and trigram (phrases containing three words) words and consider them as one word.

After the preprocessing step, we applied a commonly used term-weighting method called TF-IDF, which is a pre-filtering stage with all the included TM methods. TF-IDF is a numerical statistic measure used to score the importance of a word (term) in any content from a collection of documents based on the occurrences of each word, and it checks how relevant the keyword is in the corpus. Also, it not only considers the frequency but also induces discriminative information for each term. Term frequency represents how many times a word appears in a document, divided by the total number of words in that document, while inverse document frequency calculates how many documents the term appears in and divides it by the number of documents in the corpus. Furthermore, calculating the TF-IDF weight of a term in a particular document requires calculating term frequency [TF(*t, d*)], which is the number of times that the word *t* occurred in document *d*; document frequency [DF(*t*)], which is the number of documents in which term *t* occurs at least once; and inverse document frequency (IDF), which can be calculated from DF using the following formula. The IDF of a word is considered high if it occurred in a few documents and low if it occurred in many documents (Ahmed Taloba et al., 2018). The TF-IDF model is defined in Equations (2) and (3):

(2) $$\begin{array}{r} \text{TF }=\;\frac{\text{num of occurrences of word in documents }}{\text{ num of words in all documents }} \end{array}$$

(3) $$\begin{array}{r} \text{IDF }=\text{ log}\frac{\text{num of documents }}{\text{ num of documents with word occurs }} \end{array}$$

## Evaluation

### Evaluation Procedure

OSNs include a huge amount of UGC with many irrelevant and noisy data, such as non-meaningful, inappropriate data and symbols that need to be filtered before applying any text analysis techniques. In our work, we deal with text mining subjects. This is quite difficult to achieve since the objective is to analyze unstructured and semi-structured text data. Without a doubt, employing methods that are similar to human–human interaction is more convenient, where users can specify their preferences over an extended dialogue. Also, there is a need for further effective methods and tools that can aid in detecting and analyzing online social media content, particularly for those using online UGC as a source of data in their systems. We implemented the Gensim toolkit due to its ease of use and because it gives more accurate results. Gensim was the most popular tool used in many recent studies, and it offers more functionality; it also contains an NLP package that has effective implementations of several well-known functionalities for the TM methods such as TF-IDF, LDA, and LSA.

In our experiment, we tested numerous TM methods on commonly used public text dataset for experiments in the text application task called the 20-newsgroup data and short conversation data from the Facebook social network site, as shown in Table 3.

**Table 3:** Statistics of our involved datasets.

**Dataset**	**Description**
20-newsgroup^1^ data	20,000 documents Average document length: 28 Topics: computer, recreation, science, miscellaneous, politics, and religion as distinct classes
Facebook conversations^2^	20 text conversations Approximately 87 sentences and 7,250 words. Topics: travel, food, restaurant, hotel booking, flight booking, study and university

1 *http://people.csail.mit.edu/jrennie/20Newsgroups/*.

2 *https://github.com/Rania2016/20-FACEBOOK-CONVERSATIONS*.

We evaluate the topic quality and performance of five frequently used TM methods. In addition, we calculate the statistical measures precision, recall, and *F*-score to assess the accuracy verification within a different number of features *f*, *f* = 10, 100, 1,000, 10,000. Besides, it is important to consider how many topics we want to extract and find in the corpus, and this step must be decided by a human user. We ran an experiment and create four extracted topics *t, t* = 5, 10, 20, and 50. Recall, precision, and *F*-score calculations are presented in Equations (4–6), respectively.

- Recall (*R*) is a common information retrieval metric that measures the fraction of relevant items among the recommended items.
- Precision (*P*) is a common information retrieval metric that measures the fraction of retrieved recommended items to the actual relevant items.
- The *F*-score (*F*) measures the effectiveness of the retrieval and is calculated by combining the two standard measures in text mining, namely, recall and precision.

(4) $$\begin{array}{r} \text{Recall}=\frac{tp}{tp+fn} \end{array}$$

(5) $$\begin{array}{r} \text{Precision}=\frac{tp}{tp+fp} \end{array}$$

(6) $$\begin{array}{r} \text{F}-\text{score}=\frac{Precision\;.\;Recall}{Precision+Recall} \end{array}$$

Note that the true positive (TP) is the number of keywords detected as a topic, the false positive (FP) is the number of non-keywords detected as a topic, the true negative (TN) is the number of non-keywords detected as non-topics, and the false negative (FN) is the number of topics detected as non-topics.

### Data Extraction and Experiment Results

In our data extraction stage, we aim to extract topics from clusters of input data. As we mentioned before, we did our second evaluation several times by applying a different number of features *f* and topics *t, f* = 10, 100, 1,000, and 10,000 and *t* = 5, 10, 20, and 50. Tables 4–6 present our initial results of the topic performance and accuracy after applying some common standard metrics that are applicable to the TM methods, related to the 20-newsgroup data.

**Table 4:** Performance of involved topic modeling methods with different extracted topics *t, t* = 5 and 10, (average value of recall, precision, and *F*-score).

**TM method**	**Number of topics**					
	**5**			**10**		
	***R***	***P***	***F***	***R***	***P***	***F***
LSA	0.1546419	0.1501913	0.1523841	0.1825729	0.1838501	0.1881104
LDA	0.150000	0.1533333	0.1511765	0.1238715	0.1067887	0.1146975
NMF	0.2577005	0.2522465	0.2549443	0.4734466	0.4791113	0.4762621
PCA	**0.3860860**	**0.3878723**	**0.3869771**	**0.5546999**	**0.5616488**	**0.5581528**
RP	0.1137931	0.1105053	0.1121251	0.1156499	0.1123152	0.1139581

*Bold values represent the highest performance results*.

**Table 5:** Performance of involved topic modeling methods with different extracted topics *t, t* = 20 and 50 (average value of recall, precision, and *F*-score).

**TM method**	**Number of topics**					
	**20**			**50**		
	***R***	***P***	***F***	***R***	***P***	***F***
LSA	0.2198939	0.2128799	0.2163301	0.2210345	0.2279532	0.2294835
LDA	0.3446734	0.3435585	0.3489088	0.2312177	0.2174433	0.2336483
NMF	0.5918747	0.5977849	0.5948151	0.6915826	0.6952324	0.6934027
PCA	**0.6339618**	**0.6392421**	**0.6365910**	**0.7044610**	**0.7086668**	**0.7065576**
RP	0.1132626	0.1106185	0.1119249	0.1084881	0.1052548	0.1068470

*Bold values represent the highest performance results*.

**Table 6:** Performance of involved topic modeling methods with a different number of features *f*, *f* = 10, 100, 1,000, and 10,000 (average value of recall, precision, and *F*-score).

**TM method**	**Number of features**			
	**10**	**100**	**1,000**	**10,000**
	***F*-score**	***F*-score**	***F*-score**	***F*-score**
LSA	0.108238987	0.177539633	0.196284973	0.187135878
LDA	0.118222579	**0.313004427**	**0.596767795**	**0.616768865**
NMF	**0.124100619**	0.246607097	0.384831984	0.478534632
PCA	0.118742505	0.273855576	0.459150019	0.553060479
RP	0.123841731	0.101052719	0.126599635	0.114772128

*Bold values represent the highest performance results*.

We observe that each TM method we used has its own strengths and weaknesses, and during our evaluation, the results of all the methods performed similarly. Briefly, by comparing the outcomes of the extracted topics, PCA produced the highest term–topic probability; NMF, LDA, and LSA models provided similar performance; and RP statistical scores were the worst compared to other methods. The probabilities range from 0 to 1 in all evaluated TM methods. However, it provided a selection of non-meaningful words, like domain-specific stop words that are not suitable for further processing. Also, we notice that LDA methods provide the best learned descriptive topics compared to the other methods, aside from some methods that failed to create topics that aggregate related words, like the LSA TM method which usually performs best at creating a compact semantic illustration of words in a corpus. In addition, in Tables 4–6, PCA and RP methods had the best and worst statistical measure's results, respectively, when compared to other TM with similar performance results. However, PCA and RP methods distributed random topics that made it hard to obtain the main-text main topics from them.

**Figure 6:**
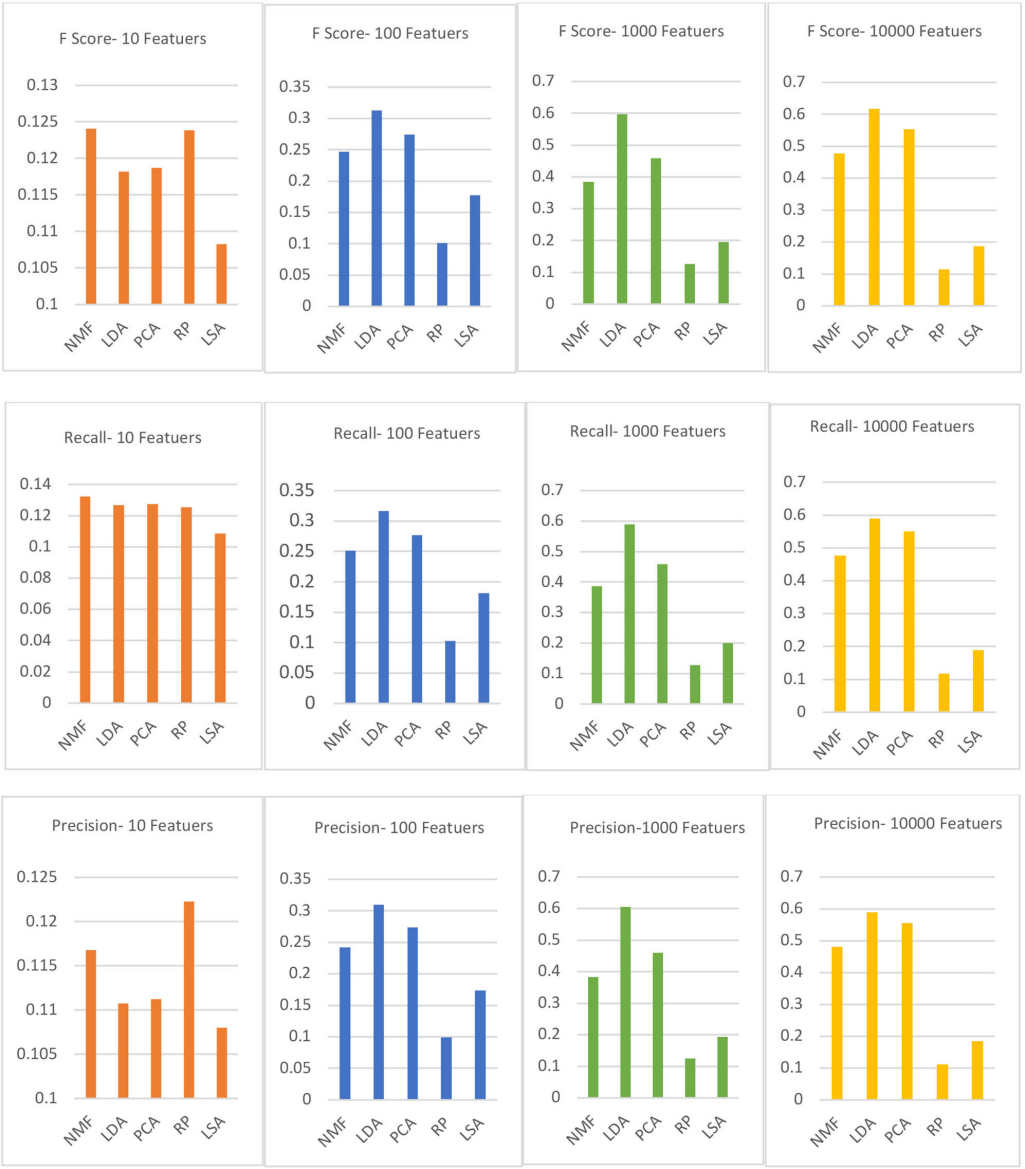
The *F*-score average results with different numbers of features *f* = 10, 100, 1,000, 10,000 (20-newsgroup dataset).

Moreover, the LDA and NMF methods produce higher-quality topics and more coherent topics than the other methods in our evaluated Facebook conversation dataset, but the LDA method was more flexible and provided more meaningful and logical extracted topics, especially with fewer numbers of topics that match our final aim of defining a TM method that can understand the online UGC. Also, when comparing LDA and NMF methods based on their runtime, LDA was slower, and it would be a better choice to apply NMF specifically in a real-time system. However, if runtime is not a constraint, LDA outperforms the NMF method. NMF and LDA have similar performances, but LDA is more consistent. The dataset provided in our experiment tested over a certain number of topics and features, though additional investigation would be essential to make conclusive statements. Also, we ran all the topic methods by including several feature numbers, as well as calculating the average of the recall, precision, and *F*-scores. As a result, the LDA method outperforms other TM methods with most features, while the RP model receives the lowest *F*-score in most runs in our experiments. The graphs in Figure 6 present the average results of *F*-scores with a different number of feature *f* on the 20-newsgroup dataset. Aside from the TM method comparison, the graphs show that a higher *F*-score was obtained with the LDA model. In addition, over the Facebook conversation data, the LDA method defines the best and clearest meaning compared to other examined TM methods.

Moreover, we measured the topic coherence score, and we observed that extracting fewer numbers of keywords led to a high coherence score in LDA and NMF TM methods. As a result, obtaining fewer keywords can help define the topic in less time, which is useful for our future developing real-time social recommendation system which aims to analyze the user's online conversation and deliver a suitable task such as advertainment. Based on our experiments, we decided to focus on LDA and NMF topic methods as an approach to analyze short social textual data. Indeed, LDA TM is a widely used method in real-time social recommendation systems and one of the most classical state-of-the-art unsupervised probabilistic topic models that can be found in various applications in diverse fields such as text mining, computer vision, social network analysis, and bioinformatics (Vulić et al., 2015; Liu et al., 2016).

## Conclusion

The internet assists in increasing the demand for the development of business applications and services that can provide better shopping experiences and commercial activities for customers around the world. However, the internet is also full of information and knowledge sources that might confuse users and cause them to spend additional time and effort trying to find applicable information about specific topics or objects. Conversely, the need to analyze short texts has become significantly relevant as the popularity of microblogs such as Twitter grows. The challenge with inferring topics from short text is due to the fact that it contains relatively small amounts and noisy data that might result in inferring an inaccurate topic. TM can overcome such a problem since it is considered a powerful method that can aid in detecting and analyzing content in OSNs, particularly for those using UGC as a source of data. TM has been applied to numerous areas of study such as Information Retrieval, computational linguistics and NLP. Also, it has been effectively applied to clustering, querying, and retrieval tasks for data sources such as text, images, video, and genetics. TM approaches still have challenges related to methods used to solve real-world tasks like scalability problems.

This paper delved into a detailed description of some significant applications, methods, and tools of topic models, focusing on understanding the status of TM in the digital era. In our evaluation, we used two textual datasets: the 20-newsgroup data and short conversation data from the Facebook social network site. The performances achieved by TM methods were compared using the most important and common standard metrics in similar studies, namely, recall, precision, *F*-score, and coherence. We also defined which methods can deliver maximum well-organized and meaningful topics. As a result, we found that all of the included TM methods we used to have much in common, like transforming text corpora into term–document frequency matrices and using the TF-IDF model as a prefiltering model, producing topic content weights for each document and other processes. Despite these similarities, the two TM methods that generated the most valuable outputs with diverse ranges and meanings were the LDA and NMF TM methods. The work presented in this paper can be a vital reference for researchers on short-text TM.

## Data Availability Statement

Publicly available datasets were analyzed in this study. This data can be found here: http://people.csail.mit.edu/jrennie/20Newsgroups/.

## Author Contributions

TY and MB contributed to the design of the research, and to the writing of the journal. All authors contributed to the article and approved the submitted version.

## Conflict of Interest

The authors declare that the research was conducted in the absence of any commercial or financial relationships that could be construed as a potential conflict of interest.

## Footnotes

^1^https://pypi.org/project/gensim/

^2^https://nlp.stanford.edu/software/tmt/tmt-0.4/
